# Antibody Microarray Immunoassay for Simultaneous Quantification of Multiple Mycotoxins in Corn Samples

**DOI:** 10.3390/toxins10100415

**Published:** 2018-10-15

**Authors:** Xian Zhang, Zuohuan Wang, Yun Fang, Renjie Sun, Tong Cao, Narayan Paudyal, Weihuan Fang, Houhui Song

**Affiliations:** 1 China-Australian Joint Laboratory for Animal Health Big Data Analytics, Zhejiang Provincial Engineering Laboratory for Animal Health Inspection and Internet Technology, College of Animal Science and Technology, Zhejiang A&F University, Lin’an 311300, Zhejiang, China; zhangxian073@163.com (X.Z.); whfang@zju.edu.cn (W.F.); 2 Zhejiang University Institute of Preventive Veterinary Medicine and Zhejiang Provincial Key Laboratory of Preventive Veterinary Medicine, 388 Yuhangtang Road, Hangzhou 310058, Zhejiang, China; zuohuanwang@zju.edu.cn (Z.W.); rjsun@zju.edu.cn (R.S.); caotong0915@zju.edu.cn (T.C.); narayan.paudyal@outlook.com (N.P.); 3 Technic Center of Zhejiang Entry-Exit Inspection and Quarantine Bureau, 126 Fuchun Road, Hangzhou 310012, Zhejiang, China; fy@yw.ziq.gov.cn

**Keywords:** mycotoxins, biotin-streptavidin, microarray, quantification

## Abstract

We developed and tested a prototype of an antibody microarray immunoassay for simultaneous quantitative detection of four typical mycotoxins (aflatoxin B_1_, ochratoxin A, zearalenone, and fumonisin B_1_) in corn samples. The test kit consisted of a nitrocellulose membrane layered with immobilized monoclonal antibodies against mycotoxins. During the assay, the mycotoxin-protein conjugates were biotinylated. The signal detection was enhanced by a combination of the biotin-streptavidin system and enhanced chemiluminescence (ECL). This improved the sensitivity of the assay. Under the optimized conditions, four calibration curves with goodness of fit (*R*^2^ > 0.98) were plotted. The results showed that the detection limits for aflatoxin B_1_, ochratoxin A, zearalenone, and fumonisin B_1_ were 0.21, 0.19, 0.09, and 0.24 ng/mL, with detection ranges of 0.47–55.69, 0.48–127.11, 0.22–31.36, and 0.56–92.57 ng/mL, respectively. The limit of detection (LOD) of this antibody microarray for aflatoxin B_1_, ochratoxin A, zearalenone, and fumonisin B_1_ in corn was 5.25, 4.75, 2.25, and 6 μg/kg, respectively. The recovery rates from the spiked samples were between 79.2% and 113.4%, with coefficient of variation <10%. The results of the analysis of commercial samples for mycotoxins using this new assay and the liquid chromatography-tandem mass spectrometry (LC-MS/MS) were comparable and in good agreement. This assay could also be modified for the simultaneous detection of other multiple mycotoxins, as well as low-weight analytes, hazardous to human health.

## 1. Introduction

Mycotoxins, the secondary metabolites produced by fungi such as *Aspergillus*, *Fusarium*, and *Penicillium*, often contaminate agricultural produce during harvest, storage, or processing [1]. The most frequently encountered and studied mycotoxins in cereal grains and feeds are aflatoxins, ochratoxins, zearalenone, and fumonisins [2]. The major toxicities of these mycotoxins include carcinogenicity (aflatoxin B_1_, AFB_1_), potentially carcinogenic and nephrotoxic agents (ochratoxin A, OTA), estrogenic and reproductive toxicity (zearalenone, ZEN), and equine leucoencephalomalacia and porcine pulmonary edema (fumonisin B_1_, FB_1_) [3]. Their widespread distribution has become a major concern in food and feed safety, and simultaneous occurrence of multiple mycotoxins is a common phenomenon. Synergism in toxicities is observed when a multiplex of mycotoxins occurs in a single product [4,5]. Considering the serious health risk of mycotoxins, many countries and regulatory authorities, such as the Joint Food and Agriculture Organization (FAO)/World Health Organization (WHO) Expert Committee on Food Additives (JECFA), have defined the maximum residue limit (MRL) in food products at the ppb level (parts per billion or μg/kg) [6].

Series’ of analytical methods have been developed and are in use to quantify the levels of these mycotoxins. Instrumental methods, such as high-performance liquid chromatography (HPLC) [7], liquid chromatography-tandem mass spectrometry (LC-MS/MS) [8], and gas chromatography-mass spectrometry (GC-MS) [9] are highly sensitive and produce reliable results. However, these require complex and tedious sample preparatory steps, and are costly while also being time-consuming. Immunoassays, such as multiplex flow cytometric immunoassay [10], microarray-based methods [11], and fluorescence polarization immunoassay [12], have proven to be excellent methods for multi-component detection due to their advantage of high throughput. The need for special instruments and skilled technicians in the detection process restricts their extensive use. Traditional immunoassay, the enzyme-linked immunosorbent assay (ELISA), has been widely used as a screening method due to its high sensitivity, speed, and low-cost, but is possibly difficult to implement as a high-throughput detection method. Promising methods, such as series electrochemical assays, have been employed for the detection of mycotoxins, but significant improvements still need to be made on some key components (e.g., simplicity, ease of operation, and multi-mycotoxin recognition) to render them an ideal analytical platform in the near future [13]. The need for methods able to detect multiple-mycotoxin in one assay is the background on which this study is based.

Since the development of arrays, this technology has been widely applied in many areas, such as clinical medicine, cell biology, proteomics, and analytical chemistry [14]. Several suspension arrays that use an indirect competition strategy have been reported to be used for detection of multiple mycotoxins [15,16,17]. These methods use lengthy, complex procedures, and reactions are carried out in Eppendorf tubes. Solid-phase array is a combination of immunoassay and protein microarrays. In this system, signal responses produced by antigen (Ag)-antibody (Ab) interaction is observed by a confocal laser scanner and image extraction software. This high-throughput detection platform has the advantages of sensitivity, rapidity, and accuracy, and has great potential for applications in agriculture and food chemicals, such as pesticides, pathogens, antibiotics, and mycotoxins [18,19,20,21]. However, an antibody microarray immunoassay based on the direct competition strategy and the biotin-streptavidin signal amplification system for multiple mycotoxins detection has not yet been reported in the available literature.

**Figure 1:**
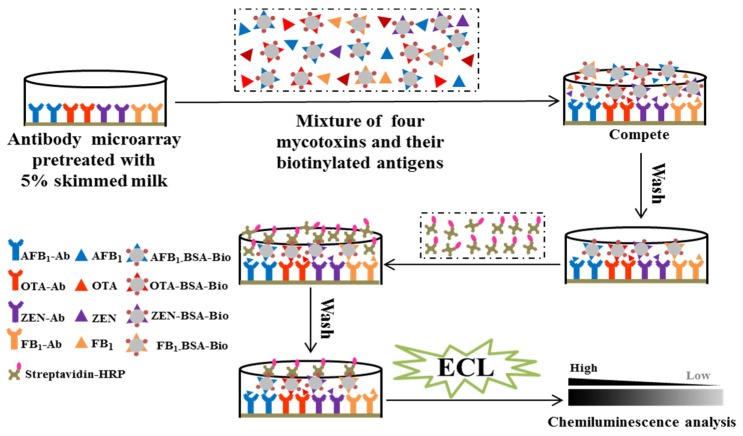
Schematic illustrations of the reaction process of the antibody microarray immunoassay.

Hence, we report a new solid-phase array strategy by combined use of the biotin-streptavidin system and ECL chemiluminescence for multiple mycotoxins detection. The schematic diagram of the antibody-based microarray is shown in Figure 1. Our results show that this method is easy to perform, can be done quickly, and provides an excellent platform for the rapid detection of multiple low molecular weight analytes, with high-throughput, accuracy, and reproducibility.

## 2. Results and Discussion

The objective of this research was to develop an antibody microarray immunoassay that can be used for the simultaneous quantitative detection of four different mycotoxins. Firstly, a design was planned, followed by prototype development for multiplexed detection, and finally, the prototype was tailored to an appropriate reaction device to facilitate signal acquisition and data analysis.

The first task of prototype development is addressed in Section 2.1 and Section 2.2. A study of the specificity among Ag-Ab pairs, and the sensitivity for multiplex detection of AFB_1_, OTA, ZEN, and FB_1_, is presented in Section 2.3 and Section 2.4. Finally, the feasibility of using this detection method to analyze commercial cereal and feed samples pre-treated with a simple extraction process, is presented in Section 2.5 and Section 2.6.

### 2.1. Development of Concept for Multiplex Antibody Microarray Immunoassay

A schematic diagram of this two-step antibody microarray immunoassay for simultaneous detection of multiple mycotoxins is shown in Figure 1. Antibodies specific to four various mycotoxins were immobilized on different detection positions of each microarray. If a certain concentration of a target mycotoxin is present in the test sample, the mycotoxin will compete with the corresponding biotinylated mycotoxin conjugate for the binding sites on the specific antibodies immobilized on the nitrocellulose (NC) membranes. Hence, a high concentration of the target mycotoxin will result in a low density of biotinylated mycotoxin conjugates captured by its specific antibody. After incubation with Streptavidin-horseradish peroxidase (Strep-HRP) and the Enhanced Chemiluminescence (ECL) substrate, a colorimetric signal that is inversely proportional to the concentration of target mycotoxin is generated.

A set of five dots immobilized with serial concentrations of Strep-HRP (0.0625, 0.125, 0.25, 0.5, and 1 ng/mL) were used as an internal standard curve in each microarray. This provided a base signal to normalize the variable uniformity coefficient of the ECL substrate and exposure efficiency arising due to imaging system. Variability caused by the differences in the amount of ECL substrate and exposure position can be compensated for by using these internal controls as mentioned above. The grayscale in each spot was quantified using Lux Scan 3.0 software, which has the feature of optimum circle-spot auto alignment, to simplify the signal acquisition.

### 2.2. Preparation of the Hardware Prototype for Antibody Microarray Reaction

**Figure 2:**
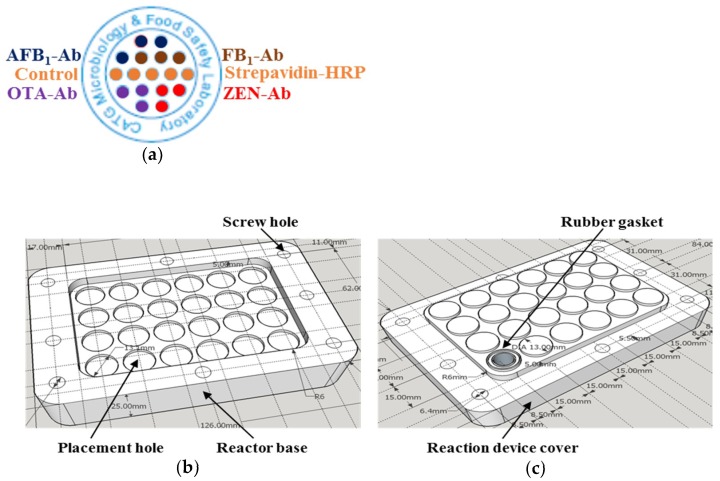
Schematic illustration of the antibody microarray and reaction hardware device for antibody microarray immunoassay. (**a**) Layout of four different monoclonal antibodies and Streptavidin-horseradish peroxidase (Strep-HRP, as control) on the antibody microarray. (**b**) Reactor base. (**c**) Reaction device cover.

Each antibody microarray included 17 isolated dots, and the reaction device could be divided into two main sections: A base with reaction wells and a cover for fixing the microarray, and the reaction buffer. A small size microtiter plate, like the commonly available 96 well plate, was chosen to make the assay simple and convenient to handle. Schematic illustrations of the antibody microarray and reaction hardware device for antibody microarray immunoassay are shown in Figure 2.

### 2.3. Evaluation of Specificity Among Ag-Ab Pairs for the Antibody Microarray

Four mycotoxin antibodies were characterized and screened using competitive indirect enzyme-linked immunosorbent assay (iELISA) [19]. Monoclonal antibodies against OTA and ZEN had the same features as those characterized previously [22,23]. The cross-reactivities of mAb-AFB_1_ with the aflatoxin B_1_ analogues (aflatoxin B_2_, aflatoxin G_1_, and aflatoxin G_2_) were 3.9, 7.3, and 5.3%, respectively. Cross-reactivity of mAb-FB_1_ against FB_2_ was 11.3%. The concentration of each mycotoxin antibody and biotinylated mycotoxin conjugate was optimized for the best IC_50_ along with the value of MGImax/IC_50_ (MGImax: maximum median grayscale intensity).

Lower IC_50_ indicates better sensitivity and is usually used in immunoassay assay. Higher MGImax/IC_50_ indicates higher grayscale intensity and sensitivity, which provides a useful parameter to assess the influence of different factors on microarray performance [24]. The optimization results are shown in Table 1.

The specificity among these four biotinylated mycotoxin conjugates and antibodies is a key property of the antibody microarray immunoassay. The cross-reactivities (CRs), a parameter used for evaluating specificity, were determined using the corresponding changes in median grayscale intensity (MGI) through the microarray immunoassay, as mentioned in other immunochip methods [21]. Apart from the corresponding Ag-Ab at 100%, all the other CRs were calculated using the ratio of MGIs between the two hybridization signals.

For example, the CR value between biotinylated AFB_1_-BSA (AFB_1_-BSA-Bio) and OTA-Ab was calculated using the Formula below:

(1) $$\mathrm{CR}(\%)=\frac{\mathrm{MGI}\;\left({\mathrm{AFB}}_{1}\mathrm{-BSA-Bio}\right)\;\mathrm{and}\;\left(\mathrm{OTA-Ab}\right)}{\mathrm{MGI}\;\left(\mathrm{OTA-OVA-Bio}\right)\;\mathrm{and}\;\left(\mathrm{OTA-Ab}\right)}\times 100\%$$

**Figure 3:**
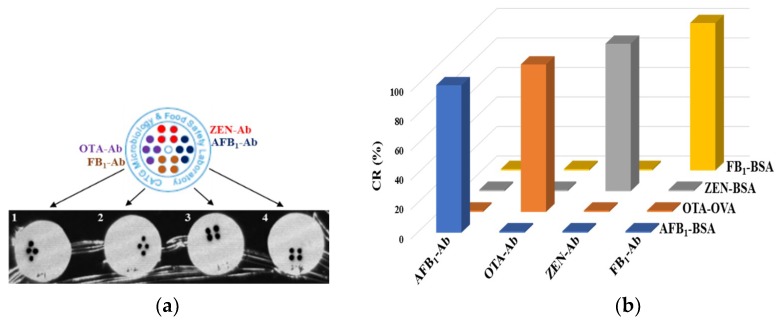
Specificity among the four antibodies and biotinylated antigens. (**a**) The hybridization image scanned from the four Abs and biotinylated antigens. (**b**) Cross-reactivities (CRs%) among the four antibodies and biotinylated antigens.

The results given in Figure 3 show that cross-reaction among these antibodies and biotinylated mycotoxin antigens is quite low (<1%), which agrees with our previously reported findings [22,23]. This confirms that these four mycotoxins can be detected simultaneously and distinguishably by using this multiple mycotoxin microarray method.

### 2.4. Calibration Curves for AFB_1_, OTA, ZEN, and FB_1_ Detection

**Figure 4:**
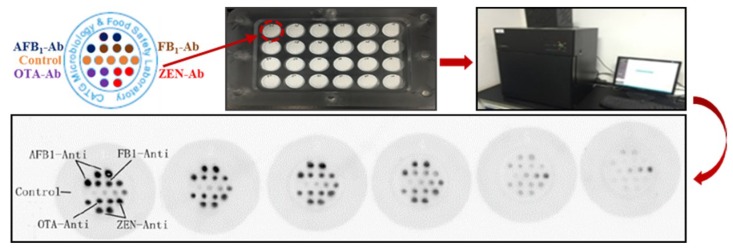
The scanned images of competitive hybridization of the four antibodies and biotinylated antigens, as well as controls.

**Figure 5:**
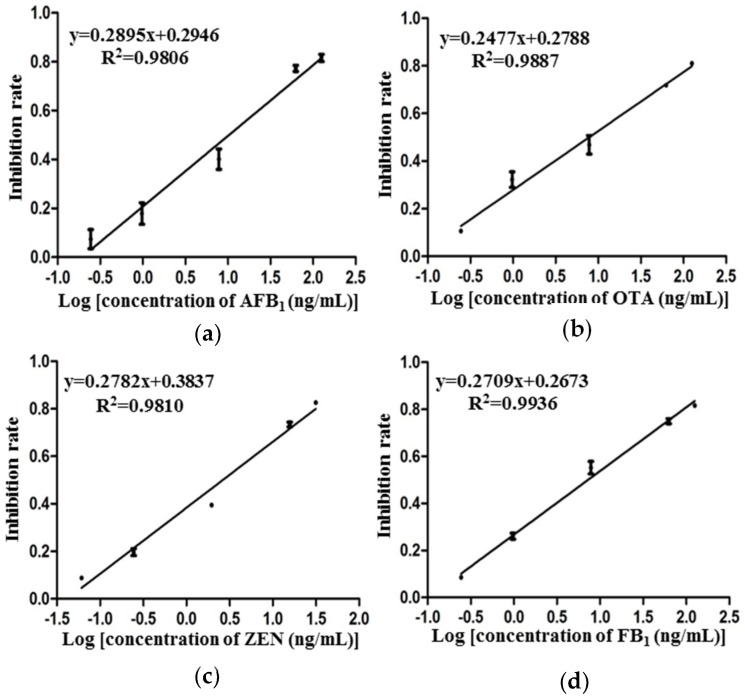
Standard curves of four mycotoxins in antibody microarray immunoassay. The log concentration of mycotoxins ((**a**): AFB_1_, (**b**): OTA, (**c**): ZEN, (**d**): FB_1_) is on the *x*-axis, while the inhibition rate is on the *y*-axis. Error bars indicate the standard deviation (data collected from three replicates).

After confirming the absence of cross-reactivities in this assay, calibration curves for each mycotoxin were measured. The standard solution is a mixture of the four mycotoxins at six various concentrations. For each target mycotoxin, a calibration curve was adjusted for the data, and a logarithmic response was obtained. The scanned images of the competitive hybridization are shown in Figure 4, and four calibration curves under the optimum conditions were calculated (Figure 5).

The limits of detection (LODs), half inhibition values (IC_50_), and linear detection ranges were calculated (Table 2). LOD is defined as the signal’s average concentration corresponding to three standard deviations from the average signals of mycotoxin-free samples [25]. A linear detection range is calculated as the concentration of target mycotoxin leading to 20–80% inhibition [26]. Results demonstrate that these four different mycotoxins could be detected simultaneously and quantitatively on one single antibody microarray.

### 2.5. Recovery Studies

Recovery experiments were performed on corn samples. Corn samples free of the four mycotoxins (AFB_1_, OTA, ZEN, and FB_1_) were spiked with various standard concentrations of AFB_1_, OTA, ZEN, and FB_1_ mycotoxins. The spiked concentrations of each mycotoxin and the results detected by using the assay are shown in Table 3. The recovery rates of the four mycotoxins were between 79.2% and 113.4%, with a relative standard deviation ranging from 4.7% to 9.1%. This data indicates that this multiple microarray chip detection assay is accurate and has good reproducibility.

### 2.6. Commercial Samples Analysis

Fifty-six dry commercial cereal samples (corn, wheat, and feed samples) were analyzed by the multiple microarray chip detection method and LC-MS/MS. Each sample was tested in triplicate to calculate standard deviation. The results are shown in Table 4 (only positive samples are listed). These results showed that the two methods have a good correlation (r = 0.93, *p* < 0.01) when evaluated by Pearson correlation (SPSS, version 19.0).

## 3. Conclusions

The results obtained in this study (samples positive to more than one mycotoxin) confirmed that the analytical method developed in this study, targeting different mycotoxins for simultaneous detection, is possible and workable. In this method, an antibody microarray strategy based on direct competition and the biotin-streptavidin signal amplification system for the simultaneous detection of four mycotoxins, is implemented. The antibody microarray immunoassay prepared here is a solid-phase array, which when compared with the liquid-phase chip assay, can be run without the need for expensive detection equipment and signal analysis software.

The combined use of the biotin-streptavidin signal enhancing system and ECL chemiluminescence improves the sensitivity of this assay. LOD is an important parameter to reflect the sensitivity of mycotoxin detection methods. For example, the LOD of AFB_1_ was 14 ng/mL using fluorescent sensor systems [27], and 1.6 ng/mL using aptamer-based fluorescent assay [28]; the LOD of OTA was 8.7 nM using nitrogen doped carbon dots and the silver nanoparticles based fluorescence method [29], 0.40 ng/mL by lateral flow strip based aptasensor [30], and 2.57 ng/mL using a novel biosensor platform [31]; the LOD of ZEN was 0.114 ng/mL using an indirect competitive enzyme-linked immunosorbent assay [32], 3.2 ng/mL using a novel recombinant cell fluorescence biosensor [33], and 0.5 ng/mL using aptamer-based fluorescence assay [34]; and the LOD of FB_1_ was 11.1 ng/mL using microarray-based immunoassay with synthetic mimotopes [35], and 1.15 ng/mL by indirect ELISA [36]. The LODs of the current assay (AFB_1_: 0.21 ng/mL, OTA: 0.19 ng/mL, ZEN: 0.09 ng/mL, and FB_1_: 0.24 ng/mL) are relatively low, which justifies its higher sensitivity. The LODs of various detection methods are shown in Table 5. When compared with other multiple detection methods, such as multicolor immunochromatographic assay [37], suspension array immunoassay [14], and lateral flow dual immunoassay [38], this new detection method also has advantages in sensitivity.

Highly sensitive assays for the detection of AFB_1_, OTA, ZEN, or FB_1_ have been established by other researchers [39,40,41,42,43,44], but for the detection of co-existing mycotoxins, multiple quantitative test assays, such as the microarray immunoassay described herein, will be more efficient at satisfying the need of detection. The assay we devised can detect four different mycotoxins in one single run, which is a pivotal advantage of this new method.

Compared with other multi-mycotoxin detection methods [15,17], this antibody microarray costs less and is easier. The direct competition procedure involves only three steps, and the reactions can be carried out in the reaction well in less than 2 h. This direct competition strategy is feasible for commercial applications since the antibodies have already been immobilized in the NC membranes.

Further work is currently being undertaken to integrate more than four targets on a single microarray. The results, if successful, will be a powerful tool to monitor mycotoxins and other small molecules in food and the environment. This study serves as a reliable basis for devising new platforms to detect other multiple mycotoxins, as well as low-weight analytes, in food or feed samples to protect human and animal health.

## 4. Materials and Methods

### 4.1. Reagents

Aflatoxin B_1_ (AFB_1_), ochratoxin A (OTA), zearalenone (ZEN), and fumonisin B_1_ (FB_1_) were purchased from Sigma Chemical Co. (St. Louis, MO, USA). Monoclonal antibodies against OTA, (mAb-OTA) and ZEN (mAb-ZEN), were generated in our laboratory [22,23]. Four mycotoxin-protein conjugates (AFB_1_-BSA, OTA-OVA, ZEN-BSA, and FB_1_-BSA), and monoclonal antibodies against AFB_1_ (mAb-AFB_1_) and FB_1_ (mAb-FB_1_), were obtained from Huaan Megnech (Beijing, China). Streptavidin-horseradish peroxidase (Strep-HRP) was obtained from Anaspec (Fremont, CA, USA). The nitrocellulose (NC) membranes (Millipore HATF 00010) were from Millipore (Bedford, MA, USA). Tween-20 was from Sangon Biotech (Shanghai, China). The EZ-Link sulfo-NHS-LC-Biotinylation Kit and SuperSignal West Pico chemiluminescent substrate were purchased from Thermo Fisher Scientific (Wyman Street, Waltham, MA, USA). Other reagents of analytical grade, as required, were purchased from Sinopharm Chemical Reagent Co. Ltd. (Shanghai, China). Four mycotoxins (AFB_1_, OTA, ZEN, and FB_1_)-free corn samples (as confirmed by LC-MS/MS), as well as commercial cereal samples (corn, wheat, and feed), were provided by the Zhejiang Entry Exit Inspection and Quarantine Bureau.

### 4.2. Apparatus

The Personal ArrayerTM 16 (TeleChem International, Sunnyvale, CA, USA) was adopted as the microarray printing robot. The Gel 3100 Chemiluminescent and Fluorescent Imaging System with Signal Analysis Software was provided by Sagecreation (Beijing, China). The horizontal shaker (Vortex 4 basic) from IKA (Staufen, Germany) was used. The antibody microarray and the reaction device was designed at our lab.

### 4.3. Biotinylation and Identification of Mycotoxin-Protein Conjugates

The ZEN-BSA was conjugated with biotin by using the EZ-Link sulfo-NHS-LC-Biotinylation Kit, as our previous study described [23]. The biotinylated derivative was purified by gel filtration on Zeba™ Spin Desalting Columns, and the level of biotinylation was measured by HABA competition assay. Biotinylation and identification of other mycotoxin conjugates (AFB_1_-BSA, OTA-OVA, and FB_1_-BSA) were prepared as ZEN-BSA-biotin.

### 4.4. Antibody Microarray and Reaction Device Design

Nitrocellulose (NC) membranes were chosen as the reacting microarray, and each microarray included seventeen isolated dots: Four different mycotoxin antibodies performed in three replicates (mAb-AFB_1_, mAb-OTA, mAb-ZEN, and mAb-FB_1_), and five control dots (Strep-HRP was coated to establish a standard curve for regulating the chip’s signal value).

The reaction device, which has twenty-four wells (six wells for calibration curves and others for sample detection) and can be used for the simultaneous quantitative detection of four different mycotoxins in eighteen samples, was designed by our lab and fabricated by Aowei Biotech (Hangzhou, China).

### 4.5. Preparation of the Antibody Microarray

The NC membrane was cut to an appropriate size (1.2 cm). The Personal Arrayer TM 16 with a SMP3 stealth micro-spotting pin was used to print the probes contiguously onto NC membrane in the spotting chamber, inside which the relative humidity was adjusted to about 30%, and the temperature was maintained at 25 °C. Probes covering each antibody (capture probe) and Strep-HRP (control) were spotted (2 μL) on the membrane carrier according to an arranged array. The antibody microarrays were then blocked with 5% (*m*/*v*) skimmed milk in TBST (0.01 M TBS containing 0.05% Tween 20 (*v*/*v*), pH 7.4) at 37 °C for 2 h to minimize the nonspecific binding, followed by thorough washing in TBST. The antibody microarrays were then dried, sealed, and stored at 4 °C for later use.

The concentration of each mycotoxin antibody and biotinylated mycotoxin conjugate was optimized by taking IC_50_ and maximum median grayscale intensity (MGImax)/IC_50_ into account.

### 4.6. Detection Procedure of the Antibody Microarray

This detection method is based on a direct competition strategy, and the procedure is detailed in Figure 1. The preparatory antibody microarray was put into the placement hole on the reaction base and fixed with the device cover using eight screws. The hole in the cover which corresponded to the placement hole provided the space for washing buffer or reagents storage. Rubber gaskets ensured no leakage of the liquid. Addition or suction of the liquid could be accomplished without opening the reaction device. Prepared sample solutions (250 μL) or standard solution (mixture of four mycotoxins at six various concentrations: AFB_1_, from 0 to 125 ng/mL; OTA, from 0 to 125 ng/mL; ZEN, from 0 to 31.25 ng/mL; FB_1_, from 0 to 125 ng/mL) were mixed with biotinylated antigens (250 μL mixture of four biotinylated mycotoxin conjugates at their optimized concentrations) and added into the reaction wells prefixed with the antibody microarrays.

The reaction device was shaken at 70 rpm for 45 min at 37 °C. During shaking, mycotoxins and biotinylated mycotoxin conjugates would compete for binding with their corresponding antibodies. After thorough washing with PBST (three times, every time for 5 min), the antibody microarray was treated with Strep-HRP solution (500 μL, 0.5 μg/mL), and the device was further incubated in a rotatory at 37 °C for 45 min. It was then washed three times and imaged in grayscale with the Gel 3100 Chemiluminescent and Fluorescent Imaging System.

### 4.7. Signal Acquisition and Data Analysis

The signal of direct competitive interaction was converted to quantitative data using LuxScan 3.0 software, which can auto-align the circular spots of the microarray. The data generated was analyzed using GraphPad Prism 6.0.

A calibration curve (*x*-axis representing the log concentration of Strep-HRP and *y*-axis, representing the value of grayscale) was used to estimate experimental variation within the same, or among various, microarrays. Each microarray had an individual calibration curve. The colors of the exposure images changed with the change in the competitive interaction signal. The signal intensity, from weak to strong, was represented as the color gradually changing from white to black, as shown in Figure 4. The changes in color could be detected by the naked eye, and the results could be read as semi-quantitative data. Four standard calibration curves generated from a set of six parallel microarrays measured the concentration of each mycotoxin.

### 4.8. Specificity Among the Four Antibodies and Biotinylated Conjugates

Prior to evaluating the sensitivity of this immunoassay in detecting these four target mycotoxins, it was essential to validate the multiplexed assay in terms of cross-reactivity (CR). The presence of non-specific mycotoxins in solution should not interfere with the binding of the specific antibody with its target mycotoxin.

The CRs among these four biotinylated mycotoxin conjugates and antibodies were evaluated using the antibody microarray, as shown in Figure 3. Four biotin-labeled mycotoxin conjugates were used at their optimized concentrations.

### 4.9. Recovery Studies

Corn samples were tested by LC-MS/MS before spiking and recovery tests to ensure that they did not contain any of the four mycotoxins (AFB_1_, OTA, ZEN, and FB_1_).

The corn samples were ground, and dried by overnight incubation in a 60 °C incubator, and 3 g samples were spiked with four mycotoxins at different concentrations. Spiked samples were shaken for 10 min and incubated at room temperature overnight. Fifteen milliliters of extraction solution (acetonitrile/water = 84:16, *v*/*v*) was added to each sample and shaken vigorously for 5 min. The samples were centrifuged at 3000× *g* for 10 min, and supernatants diluted five times in PBS to minimize the influence of matrix effects and solvents. The remaining procedure was the same as that described above. Each sample was in triplicate.

### 4.10. Detection of Commercial Samples by Antibody Microarray and LC-MS/MS.

All dry commercial cereal samples (including corn, wheat, and feed) were analyzed by antibody microarray and LC-MS/MS in parallel. Each sample was tested in triplicate to calculate standard deviation. For the detection by the antibody microarray, the commercial samples were extracted as the spiked samples. Validated procedures for LC-MS/MS were adopted as those described previously [45]. The correlation between the two methods was investigated by Pearson correlation.

## Figures and Tables

**Table 1:** Concentration of the monoclonal antibodies and biotinylated antigens used in the antibody microarray immunoassay.

Mycotoxins	Concentration of the Antibody (μg/mL)	Concentration of the Biotinylated Antigen (μg/mL)
AFB_1_	25	0.2
OTA	25	0.1
ZEN	50	0.4
FB_1_	12.5	0.4

**Table 2:** Detection characteristics of the antibody microarray immunoassay.

Mycotoxins	LOD (ng/mL)	IC_50_ (ng/mL)	Detection Range (IC_20_-IC_80_, ng/mL)	Regression Equation
AFB_1_	0.21	5.12	0.47–55.69	*y* = 0.2895*x* + 0.2946 (*R*^2^ = 0.9806)
OTA	0.19	7.82	0.48–127.11	*y* = 0.2477*x* + 0.2788 (*R*^2^ = 0.9887)
ZEN	0.09	2.62	0.22–31.36	*y* = 0.2782*x* + 0.3837 (*R*^2^ = 0.9810)
FB_1_	0.24	7.23	0.56–92.57	*y* = 0.2709*x* + 0.2673 (*R*^2^ = 0.9936)

**Table 3:** Recovery and coefficient of variances from corn samples spiked with three levels of four mycotoxins by the antibody microarray immunoassay.

Samples	Concentrations (μg/kg)		Recovery Rate (%) (Mean ± SD ^a^)	CV ^b^ (%)
	Spiked	Detected		
AFB_1_	20	17.27	86.3 ± 6.4	7.4
	40	31.68	79.2 ± 4.7	5.9
	60	54.87	91.5 ± 7.1	7.8
OTA	20	16.71	83.6 ± 8.1	9.7
	40	34.87	87.2 ± 7.6	8.7
	60	49.73	82.9 ± 6.8	8.2
ZEN	50	45.86	91.7 ± 8.9	9.7
	100	89.13	89.1 ± 5.7	6.4
	200	213.07	106.5 ± 7.3	6.9
FB_1_	200	189.27	94.6 ± 9.1	9.6
	400	427.62	106.9 ± 7.8	7.3
	800	907.21	113.4 ± 8.6	7.6

^a^ SD: Standard Deviation (*n* = 3). ^b^ CV: Coefficient of Variation.

**Table 4:** Mycotoxin levels in commercial cereal samples determined by the antibody microarray immunoassay and liquid chromatography-tandem mass spectrometry (LC-MS/MS).

Samples	Antibody Microarray (μg/kg, Mean ± SD ^a^)				LC-MS/MS (μg/kg, Mean ± SD)			
	AFB_1_	OTA	ZEN	FB_1_	AFB_1_	OTA	ZEN	FB_1_
Corn 1	- ^b^	12.31 ± 2.13	-	-	-	14.87 ± 1.21	-	-
Corn 2	-	-	95.37 ± 8.73	724.53 ± 57.29	-	-	109.14 ± 8.16	688.47 ± 32.07
Corn 3	-	-	72.85 ± 5.26	920.76 ± 64.79	-	-	85.03 ± 7.39	846.59 ± 50.61
Corn 4	-	27.64 ± 2.49	-	-	-	32.36 ± 2.14	-	-
Corn 7	-	20.86 ± 2.43	-	593.71 ± 41.15	-	25.57 ± 2.71	-	542.04 ± 38.32
Corn 11	-	22.59 ± 2.04	-	-	-	26.71 ± 2.13	-	-
Wheat 1	-	16.76 ± 1.36	-	-	-	20.99 ± 2.71	-	-
Wheat 6	-	-	29.78 ± 2.54	811.23 ± 62.15	-	-	34.21 ± 2.33	741.48 ± 40.32
Wheat 7	-	42.33 ± 3.89	-	-	-	49.55 ± 3.23	-	-
Wheat 11	8.79 ± 1.05	-	13.96 ± 1.19	-	10.11 ± 1.62	-	16.67 ± 1.35	-
Feed 2	23.85 ± 2.68	-	15.97 ± 1.34	398.92 ± 35.67	27.52 ± 1.84	-	19.35 ± 2.17	347.54 ± 26.38
Feed 3	-	-	14.69 ± 1.37	321.27 ± 28.94	-	-	17.52 ± 1.34	299.93 ± 21.15
Feed 6	-	-	11.38 ± 1.67	-	-	-	14.84 ± 1.89	-
Feed 7	-	39.41 ± 2.97	-	713.25 ± 61.68	-	45.62 ± 3.12	-	667.06 ± 48.81
Feed 11	-	-	26.72 ± 2.51	684.82 ± 46.39	-	-	30.34 ± 2.19	631.28 ± 37.43
Feed 12	-	-	21.85 ± 2.62	461.56 ± 35.73	-	-	27.03 ± 2.31	420.79 ± 22.27

^a^ SD: Standard Deviation (*n* = 3); ^b^ -: Not Detected.

**Table 5:** Comparison of the limit of detection (LOD) of microarray immunoassay and various other methods for mycotoxin detection.

Method	Target Analyte	LOD (ng/mL)	Ref.
Fluorescent sensor	AFB_1_	14	[27]
Aptamer-based fluorescent assay	AFB_1_	1.6	[28]
FRET based fluorescence	OTA	3.5	[29]
Lateral flow strip based aptasensor	OTA	0.40	[30]
Novel biosensor platform	OTA	2.57	[31]
ic-ELISA	ZEN	0.114	[32]
Aptamer-based fluorescence assay	ZEN	0.5	[34]
Microarray-Based Immunoassay	FB_1_	11.1	[35]
Ic-ELISA	FB_1_	1.15	[36]
Cell fluorescence biosensor	ZEN and DON	ZEN: 3.2	[33]
Lateral Flow Dual Immunoassay	ZEN and FB_1_	ZEN: 0.35, FB_1_: 5.23	[38]
Multicolor ICGA	AFB_1,_ ZEN, and T-2	AFB_1_: 0.5, ZEN: 2	[37]
Suspension Array	AFB_1,_ ZEN, DON, and FB_1_	AFB_1_: 0.56, ZEN: 0.51, FB_1_: 6.0	[14]
Microarray immunoassay	AFB_1,_ OTA, ZEN, and FB_1_	AFB_1_: 0.21, OTA: 0.19, ZEN: 0.09, FB_1_: 0.24	This work
